# Editorial: Transforming vaccine strategies: co-delivery systems for robust immunity and disease control

**DOI:** 10.3389/fimmu.2026.1803730

**Published:** 2026-03-10

**Authors:** Mohammad Owais, Saad Tayyab, Anshu Agrawal, Mohammad Furkan

**Affiliations:** 1 Interdisciplinary Biotechnology Unit, Aligarh Muslim University, Aligarh, India; 2 Faculty of Pharmaceutical Sciences, UCSI University, Kuala Lumpur, Malaysia; 3 School of Medicine, University of California, Irvine, Irvine, CA, United States; 4 Faculty of Life Sciences, Aligarh Muslim University, Aligarh, India

**Keywords:** immune response, immunotherapies, protein scaffold, targeted delivery, vaccine strategies

Live-attenuated and inactivated whole-pathogen vaccines have long served as the foundation of effective immunization, largely because they present antigens in their native structural and immunological context, eliciting broad and durable immune responses. However, this classical paradigm is increasingly limited when pathogens fail to induce long-term protective immunity, undergo rapid antigenic evolution, or cannot be safely cultured. In response, recombinant protein and subunit vaccines have emerged as safer and more controllable alternatives. Yet, when delivered in isolation, such defined antigens typically elicit weak and short-lived immune responses, underscoring a central challenge inmodern vaccinology: how to instruct the immune system effectively using minimal antigenic information.

Protein-based adjuvants (PBAs) and protein scaffolds have emerged as powerful solutions to this challenge. Unlike conventional adjuvants that primarily function through depot formation or nonspecific inflammation, PBAs actively engage innate immune pathways and shape downstream adaptive responses. Derived from diverse biological sources, PBAs function as immunological cues that bridge innate sensing and adaptive immunity. They engage pattern recognition receptors (PRRs), which include Toll-like receptors, C-type lectin receptors, NOD-like receptors, and RIG-I–like receptors, thereby mimicking pathogen-associated molecular patterns and initiating innate immune activation (1). This engagement induces proinflammatory cytokines such as IL-1β, IL-6, TNF-α, and IL-12, as well as chemokines that recruit and program antigen-presenting cells (APCs).

Beyond innate activation, PBAs decisively influence adaptive immune priming. By regulating co-stimulatory molecules, including B7 family members, on professional APCs, PBAs fine-tune T-cell activation and differentiation. Depending on their molecular architecture and delivery context, PBAs can bias immune responses toward humoral or cellular immunity-an essential feature for vaccines targeting intracellular pathogens and cancer. Importantly, some PBAs induce trained innate immunity through epigenetic and metabolic reprogramming of innate cells, enhancing subsequent adaptive responses and generating beneficial bystander effects. Collectively, these features position PBAs not as passive enhancers of immunogenicity, but as functional co-instructors of immune responses (2).

Vaccines and immunotherapies are therefore undergoing a conceptual transformation—from antigen-centric designs toward integrated platforms that actively shape immunity. Protein scaffolds and scaffold-inspired systems, including whole virions, nanovesicles, hydrogels, and nucleic acid frameworks, are increasingly recognized as functional co-delivery components (3). These scaffolds preserve antigen structure, regulate spatiotemporal release, and directly modulate immune microenvironments, thereby determining the quality, durability, and safety of immune responses. This paradigm is particularly relevant in the face of emerging viral variants, tumor immune evasion, immuno-senescence, and antimicrobial resistance.

The rapid development of COVID-19 vaccines provides a compelling demonstration of the importance of antigenic structure and scaffold integrity. While many first-generation vaccines targeted the ancestral Wuhan strain, the emergence of variants such as B.1.617.2 (Delta) highlighted the need for platforms capable of preserving antigenic authenticity. The whole-virion inactivated vaccine candidate CoviWall exemplifies a scaffold-driven strategy in which the inactivated virus itself functions as a multivalent protein scaffold (Dandotiya et al.). Manufactured under Good Manufacturing Practice and rigorously characterized for regulatory compliance, CoviWall preserved native viral protein conformations. Preclinical studies demonstrated robust neutralizing antibody responses, strong T-cell immunity, reduced lung viral loads, and attenuated pulmonary pathology following viral challenge. These findings reaffirm that intact structural scaffolds enhance both humoral and cellular immunity while conferring protection against severe disease (Dandotiya et al.).

Structural preservation is even more critical when targeting membrane proteins, which constitute a major class of vaccine and therapeutic targets but are notoriously difficult to exploit. Mahboob et al. studied their hydrophobic transmembrane domains, complex topology, and post-translational modifications and concluded that it often led to denaturation when removed from lipid bilayers. Recent advances in scaffold-based immunogen design—including nanodiscs, liposomes, membrane mimetics, and engineered protein frameworks—have enabled stabilization of membrane proteins in near-native environments. These scaffold-supported strategies facilitate the generation of antibodies recognizing physiologically relevant conformations and are increasingly relevant for vaccine development against pathogens and tumors that rely on membrane-associated antigens.

In cancer immunotherapy, scaffolds extend beyond antigen stabilization to active remodeling of the immune microenvironment. It has been elaborated that the cancer–immunity cycle requires efficient antigen presentation and sustained T-cell activation (Zhu et al.), yet conventional immunotherapies often suffer from poor targeting and systemic toxicity. Hydrogels have emerged as versatile platform capable of locally delivering immunomodulators providing an immunomodulatory microenvironment. With extracellular matrix–mimicking properties and tunable physicochemical characteristics, hydrogels create localized immune niches that recruit, activate, and expand immune cells. Studies in breast cancer demonstrate that hydrogel depots enhance therapeutic efficacy while minimizing adverse effects, highlighting that material properties themselves can be immunologically instructive.

A complementary example of scaffold-enabled immune modulation is provided by nanovesicle-based strategies designed to overcome tumor immune evasion (Xie et al.). In this study, it has been elucidated that tumor cells frequently downregulate major histocompatibility complex class I (MHC-I) molecules, limiting cancer vaccine efficacy. Redox-responsive nanovesicles encapsulating clinically approved tyrosine kinase inhibitors, such as sunitinib or sorafenib, selectively accumulate in tumors and activate IFN-γ/STAT1 signalling, restoring MHC-I expression. When combined with whole-tumor-antigen nano-vaccines, this co-delivery approach elicits synergistic antitumor immunity, slow tumor growth, and prolong survival in breast cancer and melanoma models. Here, the scaffold coordinate drug delivery, immune signalling, and antigen presentation.

Scaffold-based vaccination can also exert systemic immunomodulatory effects, as illustrated by studies on Orf virus vaccination. A B2L gene-based DNA vaccine has been reported to induce stronger cellular and humoral immunity than a live-attenuated vaccine and was associated with favourableremodelling of the gut microbiota. Increased beneficial bacterial populations and enhanced metabolic pathway diversity suggest that vaccine scaffolds can influence host–microbiota–immune interactions, expanding the concept of adjuvanticity beyond classical PRR signalling (Farooq et al.).

One study, conducted by Lee et al. concluded that, the importance of scaffold-enabled immune instruction has been reported to amplify in the aged host. Aging is associated with impaired antigen presentation, chronic inflammation, and dysfunctional T-cell responses, diminishing cancer vaccine efficacy. Scaffold-based platforms capable of sustained antigen release, localized immune activation, and integration of age-specific adjuvants offer promising strategies to overcome these barriers and tailor cancer vaccines for elderly population.

In a study, Hou et al. conducted a comprehensive bibliometric and visual analysis of published literature on nanovaccine-based cancer therapy to elucidate research trends and highlight future directions in this rapidly evolving field. Nanovaccine therapy has emerged as a promising frontier in immunology and personalized medicine, offering significant potential to enhance immune responses and precisely target specific diseases. Owing to their nanoscale size, nanovaccines are efficiently taken up by immune cells, resulting in robust immune activation. They can also be engineered to incorporate immune-stimulating components, thereby improving vaccine efficacy. Importantly, nanovaccines can be personalized to deliver tumor-specific antigens, enabling targeted activation of the immune system against cancer cells. A growing body of evidence has demonstrated the effectiveness and therapeutic potential of nanovaccines in cancer treatment. However, bibliometric analyses focusing on nanovaccine research in oncology remain limited.

Collectively, these studies converge on a unifying conclusion: protein scaffolds and scaffold-inspired platforms are redefining vaccines and immunotherapies as dynamic immune-engineering systems. By integrating antigen structure, targeted delivery, immune modulation, and systemic interactions, functional scaffolds transform how immunity is instructed. Embracing this paradigm will be essential for developing next-generation vaccines capable of addressing emerging pathogens, cancer immune escape, aging immunity, and antimicrobial resistance.
